# AGeS: A Software System for Microbial Genome Sequence Annotation

**DOI:** 10.1371/journal.pone.0017469

**Published:** 2011-03-07

**Authors:** Kamal Kumar, Valmik Desai, Li Cheng, Maxim Khitrov, Deepak Grover, Ravi Vijaya Satya, Chenggang Yu, Nela Zavaljevski, Jaques Reifman

**Affiliations:** DoD Biotechnology High Performance Computing Software Applications Institute, Telemedicine and Advanced Technology Research Center, U.S. Army Medical Research and Materiel Command, Ft. Detrick, Maryland, United States of America; University of Leuven, Belgium

## Abstract

**Background:**

The annotation of genomes from next-generation sequencing platforms needs to be rapid, high-throughput, and fully integrated and automated. Although a few Web-based annotation services have recently become available, they may not be the best solution for researchers that need to annotate a large number of genomes, possibly including proprietary data, and store them locally for further analysis. To address this need, we developed a standalone software application, the Annotation of microbial Genome Sequences (AGeS) system, which incorporates publicly available and in-house-developed bioinformatics tools and databases, many of which are parallelized for high-throughput performance.

**Methodology:**

The AGeS system supports three main capabilities. The first is the storage of input contig sequences and the resulting annotation data in a central, customized database. The second is the annotation of microbial genomes using an integrated software pipeline, which first analyzes contigs from high-throughput sequencing by locating genomic regions that code for proteins, RNA, and other genomic elements through the Do-It-Yourself Annotation (DIYA) framework. The identified protein-coding regions are then functionally annotated using the in-house-developed Pipeline for Protein Annotation (PIPA). The third capability is the visualization of annotated sequences using GBrowse. To date, we have implemented these capabilities for bacterial genomes. AGeS was evaluated by comparing its genome annotations with those provided by three other methods. Our results indicate that the software tools integrated into AGeS provide annotations that are in general agreement with those provided by the compared methods. This is demonstrated by a >94% overlap in the number of identified genes, a significant number of identical annotated features, and a >90% agreement in enzyme function predictions.

## Introduction

Access to inexpensive, high-throughput DNA sequencing has allowed the number of available genome sequences to grow at an exponential rate [1]. The genomes of >1,000 microbial pathogens and their near neighbors are now available, and many more are being sequenced. After a complete genome has been sequenced, there is a need to identify genomic features, such as the locations of genes that code for RNAs and proteins and positions of tandem repeats, as well as to annotate protein functions. This valuable information opens the door for new strategies in diagnostics and forensic attribution as well as for novel approaches in the identification of vaccine candidates and the discovery of “universal” drug targets through comparative genomics. For such applications, the analysis of sequenced genomes needs to be rapid, high-throughput, fully automated, integrated, and readily accessible to intended users. To address this need, we developed the Annotation of microbial Genome Sequences (AGeS) software system, which incorporates publicly available and in-house-developed bioinformatics tools and databases for integrated high-throughput genome annotation and protein function prediction.

AGeS was designed to support three main capabilities. The first is the storage of input contig sequences in FASTA format and the resulting annotation data in a central, customized database, where the data manipulation and visualization steps are performed through easy-to-use graphical user interfaces (GUIs). The second is the annotation of microbial genomes using an integrated software pipeline, which analyzes sequence contigs and locates genomic regions that code for proteins, RNAs, and other genomic elements through the Do-It-Yourself Annotation (DIYA) framework [2]. The identified protein-coding regions are then annotated using an in-house-developed high-throughput pipeline, the Pipeline for Protein Annotation (PIPA) [3]. The third capability is the visualization of annotated sequences using the open-source genome browser GBrowse [4]. To date, we have implemented full genome and protein annotation, storage, and visualization for bacterial genomes.

A few software system applications have been recently published for automated, high-quality annotation of bacterial genomes [5]–[10]. One of the first applications is the Web-based genome annotation tool BASys [5], which uses >60 annotation tools to annotate genomic features and provide protein function information. However, BASys generates enormous output files, does not integrate protein function predictions from the multiple tools, is not user friendly, and the annotation resources are not regularly updated. The RAST system [6] is another Web-based server for comprehensive genome annotation; however, its protein function annotation uses subsystem-based ontology, which cannot be easily mapped to the *de facto* standard Gene Ontology (GO) [11] annotation. In addition, many large genome annotation centers provide annotation services, such as the Annotation Engine at the J. Craig Venter Institute (JCVI) [8], the Genoscope's annotation service MicroScope [12], and the Microbial Annotation Pipeline of the Integrated Microbial Genomes system [10]. However, these Web-based annotation services may not be the best solution for researchers that need to annotate a large number of genomes, possibly including proprietary data, and store them locally for further analysis.

The integration of bioinformatics resources into pipelines for local installation is not trivial and requires significant bioinformatics expertise. While recently published integrated software systems, such as DIYA [2] and the Genome Reverse Compiler [13], provide standalone packages for genome annotation, they do not have fully integrated and automated visualization tools and do not enable the full utilization of parallel computing, which significantly limits their choice of annotation tools. AGeS attempts to address some of these limitations by providing the following functionalities to process resource-intensive, proprietary genomic sequences:

- fully integrated and automated annotation of completed and draft bacterial genomes, providing GO-based protein function annotations;
- high-throughput annotation through efficient parallelization of the various bioinformatics resources and use of high-performance computing;
- visualization based on the familiar open-source genome browser GBrowse [4] and a link to download annotated genomes in GenBank [14] format; and
- free availability of the source code.

## Methods

The AGeS system was designed and implemented to provide a standalone, integrated solution that users can install on their computers. AGeS can be installed on either a standalone Linux computer or a Linux cluster by following the step-by-step instructions provided in the User and Installation Manual (see Document S1). All bioinformatics tools integrated into AGeS are incorporated during the installation process. When run on a multi-core Linux computer or a Linux cluster, AGeS supports OpenMPI for parallel execution and PBS for batch submission.

### System architecture

**Figure 1:**
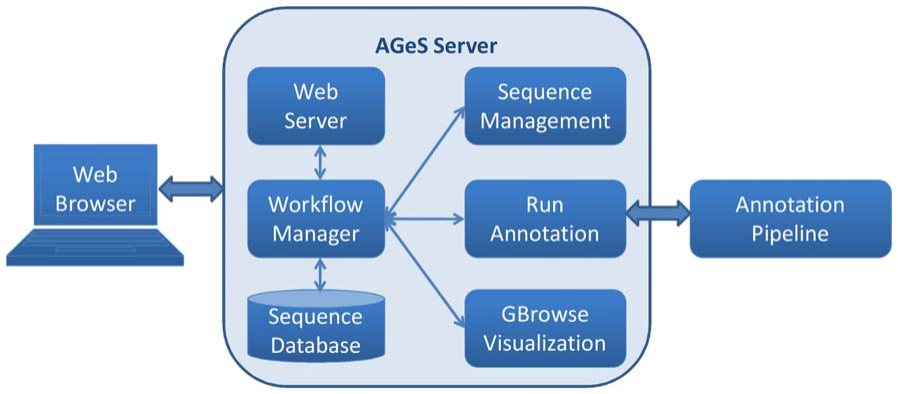
Annotation of microbial Genome Sequences (AGeS) system architecture. The Web server hosts the AGeS Web application and accepts user requests via standard Web browsers. The workflow manager handles user requests for sequence management, runs the annotation pipeline, and presents the annotation results via GBrowse visualization. The sequence database stores all sequence and job-related data.

Figure 1 shows the system architecture of AGeS. It comprises of a Web application server (AGeS server) that provides an easy-to-use GUI accessible via a Web browser, an embedded relational database management system for storing sequences and other job-related data, and a high-throughput software pipeline for the annotation of input genomes. The AGeS server and annotation pipeline can be accessed by multiple users through the AGeS GUI using standard Web browsers. The AGeS GUI provides three main functions to the users: (*i*) sequence management for uploading and manipulating genomic sequences and their properties, such as genus, species, and strain, along with optional information compliant with the Minimum Information About a Genomic Sequence [15]; (*ii*) job submission for running the annotation pipeline; and (*iii*) graphical visualization of the annotated sequence with GBrowse. As shown in Figure 1, the AGeS server uses a workflow manager module to guide the entire lifecycle of the user's job; starting from the upload of an input sequence and ending with the visualization of the annotated sequences.

The annotation pipeline is a standalone application that is initiated by the workflow manager at the user's request and runs in batch mode on a Linux cluster to achieve high throughput. The user is provided with two options for obtaining the annotation results: (*i*) bookmarking the results page and loading it back at a later time or (*ii*) providing an e-mail address for automated notification upon the completion of the annotation. AGeS is a stateful system, and all of the data relating to the user's job reside in an embedded relational database management system. A unique session is created for each new user or after a user's prior session has been terminated. After completion of the annotation, the results are automatically stored within that user's session. The annotation results can be interactively viewed using GBrowse or downloaded as a GenBank file.

The AGeS system has been designed for easy integration with future sequence analysis modules. Its Web applications use technologies based on open standards, including Java [16], J2EE [17], JavaServer Faces (JSF) [18], ICEfaces [19], asynchronous JavaScript and XML (AJAX) [20], jBPM [21], and Apache ActiveMQ [22]. The GUI has been developed using server-side Java codes that use a JSF- and AJAX-based Application Programming Interface (API) from ICEfaces, which provides a rich set of user interface components that support desktop application-like features in a Web application. The workflow manager has been implemented using the jBPM workflow engine API for controlling the execution of various modules and uses the Apache ActiveMQ server for asynchronous message passing between the modules and workflow engine. The AGeS server comes preconfigured with the Jetty Web server [23] and uses Apache Derby [24] as the embedded relational database management system (RDBMS) to provide persistence support for workflow and sequence annotation data. AGeS also supports the use of external RDBMS, such as PostgreSQL, by modifying a configuration file.

### Annotation pipeline

**Figure 2:**
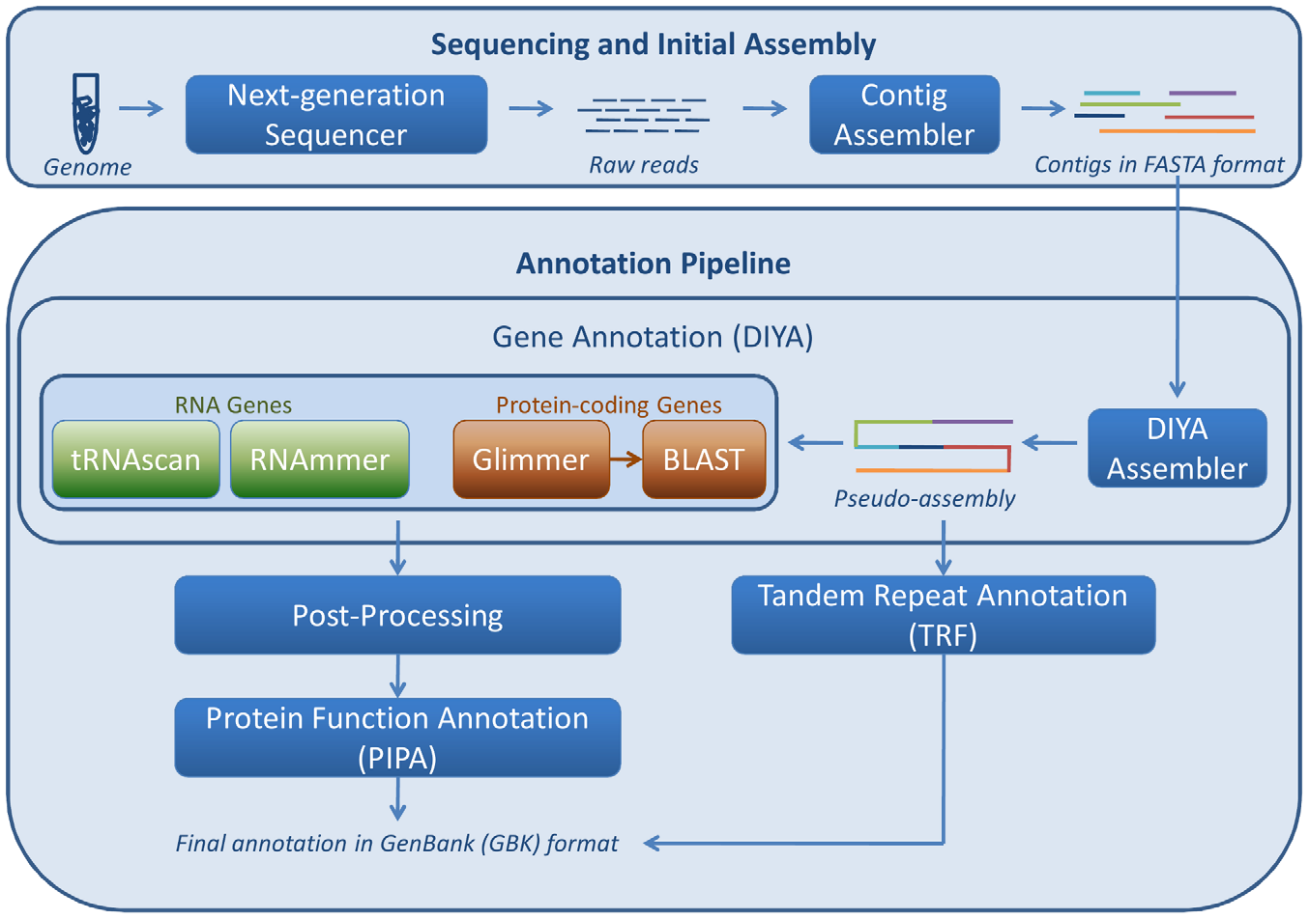
Schematic representation of the various tools of the genome annotation pipeline. Given assembled contigs in a FASTA format file, processing starts with the Do-It-Yourself Annotation (DIYA) genome annotation tool, followed by post-processing, tandem repeat annotation, and protein function prediction with Pipeline for Protein Annotation (PIPA).

As shown in Figure 2, the annotation pipeline takes as input assembled contiguous sequences, or contigs, in FASTA format files generated by high-throughput sequencing technologies [25]–[27]. AGeS uses the DIYA framework [2] to analyze input contigs. Contigs are first concatenated to create a continuous sequence, or pseudo-assembly, where a sequence of 18 bp consisting of 6 frame translational stop codons is used for filling the space between adjacent contigs.

For genome annotation, DIYA was customized to locate genomic regions that code for proteins using Glimmer [28], rRNA using RNAmmer [29], and tRNA using tRNAscan-SE [30]. Within the DIYA framework, the system uses BLAST [31] searches to extract coding regions from the Glimmer predictions and to infer gene products by transferring annotation from the best BLAST match. In addition, the system finds tandem repeats in the pseudo-assembled sequence using Tandem Repeats Finder [32]. Outputs from the different DIYA component programs are post-processed and parsed to generate a file in the GenBank format.

The identified protein-coding regions are annotated using the high-throughput protein function annotation methods implemented in PIPA [3]. One of the most useful features of PIPA is that it exploits and consistently consolidates protein function information from disparate sources, including the in-house-developed CatFam enzyme profile database [33]. An added benefit is that the consolidated function predictions are given in GO terms, which is the *de facto* standard for protein annotation. The protein annotation results from PIPA are included in the GenBank file exported from AGeS. Table 1 shows the DIYA and PIPA genome and protein annotation tools, respectively, that have been implemented into AGeS.

**Table 1:** List of genome annotation tools incorporated in DIYA and protein annotation tools integrated in PIPA.

Resource	Description	Reference
**DIYA**	**Modular and configurable bacterial genome annotation pipeline**	[2]
Glimmer	Program for microbial gene identification	[28]
RNAmmer	Program for rRNA gene prediction	[29]
tRNAscan-SE	Program to identify tRNAs	[30]
TRF	Tandem Repeats Finder	[32]
**PIPA**	**Pipeline for Protein Annotation**	[3]
CatFam	Enzyme profile databases based on three- and four-digit EC numbers	[33]
CDD	NCBI Conserved Domains Database	[48]
COG	Clusters of Orthologous Groups of proteins	[49]
InterPro	Integrated member databases	[50]
PSORTb	Prediction of bacterial subcellular localization	[51]
Phobius	A combined transmembrane topology and signal peptide predictor	[52]

DIYA, Do-It-Yourself Annotation; PIPA, Pipeline for Protein Annotation; EC, Enzyme Commission.

### Availability and Requirements

**Project name:** AGeS

**Project home page:** http://www.bhsai.org/ages.html

**Operating system:** Linux

## Results

### Software validation

We validated AGeS by comparing annotations of bacterial genomes provided by the tools integrated in AGeS with annotations from other sources. For this validation, we used (*i*) two draft genomes, *Staphylococcus hominis* SK119 and *Staphylococcus aureus* subsp. *aureus* TCH60, and (*ii*) one completed genome, *Yersinia pestis* CO92. The 2.2-Mbp *S. hominis* SK119 genome, sequenced by JCVI [34], consists of 37 contigs. The 2.8-Mbp *S. aureus* subsp. *aureus* TCH60 genome, sequenced by the Human Genome Sequencing Center at Baylor College of Medicine (BCM) [35], consists of 65 contigs. Both of these draft genomes were sequenced using 454 pyrosequencing technology [25]. The 4.6-Mbp complete *Y. pestis* CO92 genome was sequenced by the Wellcome Trust Sanger Institute [36] using Sanger sequencing technology.

We retrieved the annotations for these three genomes from the corresponding sequencing centers and re-annotated them with AGeS. These genomes were neither used in the development nor in the configuration of AGeS. Table 2 compares the annotations of important genomic features inferred by AGeS against those provided by the original annotations from the corresponding centers (the AGeS annotations in GenBank format for the draft genomes are provided in GenBank File S1 and GenBank File S2). Each of the two compared annotation sources predicted identical numbers of rRNA features for each of the three genomes and obtained similar numbers of predictions for the genes, CDSs, and tRNAs. We performed a more detailed analysis of the features predicted by AGeS by comparing their genomic locations with those predicted by the other annotation sources. For each feature, we divided the total number of AGeS predictions into the following five categories: 1) identical features; 2) identical start position only; 3) identical end position only; 4) neither start nor end position matches exactly but the features overlap; and 5) no overlap, which represents the case where the feature was not predicted by the other annotation method. Table 3 summarizes the detailed comparison of the number of genes in these five categories for the three genomes analyzed. We performed similar comparisons for CDS, tRNA, and rRNA features (data not shown). For *S. hominis* SK119, we found that >78% of the genes were identical across both predictions. Most of the remaining genes overlapped at the start or end positions, with only 0.2% of the predictions unique to AGeS. AGeS missed 24 genes (∼1%), which were only predicted by JCVI. In addition, 52 of the 53 tRNAs and 3 of the 4 rRNAs were identical. For the *S. aureus* subsp. *aureus* TCH60 genome, ∼77% of the genes were identical, with only 1% of the predictions unique to AGeS. Another 164 genes (5.8%) predicted by BCM were missing in the AGeS annotation. We found strong similarities for RNA features, as all 57 tRNAs and 3 of the 4 rRNAs were identical between the two annotation sources, whereas the only remaining rRNA gene had a common start position.

**Table 2:** Summary of genomic features predicted by AGeS and other annotation methods for two draft genomes and one completed genome.

	*S. hominis* SK119		*S. aureus* subsp. *aureus* TCH60		*Y. pestis* CO92	
Feature	AGeS	JCVI	AGeS	BCM	AGeS	Sanger Institute
Genes	2,229	2,244	2,652	2,805	4,336	4,103
CDSs	2,172	2,182	2,591	2,738	4,249	3,885
rRNAs	4	4	4	4	19	19
tRNAs	53	52	57	57	68	70
Tandem Repeats	60	NA*	123	NA*	780	NA*

AGeS, Annotation of microbial Genome Sequences; JCVI, J. Craig Venter Institute; BCM, Baylor College of Medicine; CDSs, coding sequences; NA, not applicable.

*The original source did not provide annotation for this feature.

**Table 3:** Detailed comparison of overlapping gene segments for the three analyzed genomes, displaying the number and percentage of genes in each category.

	*S. hominis* SK119		*S. aureus* subsp. *aureus* TCH60		*Y. pestis* CO92	
Category	No. of genes	Percentage	No. of genes	Percentage	No. of genes	Percentage
1) Identical	1,753	78.7	2,037	76.8	2,639	60.9
2) Identical start	252	11.3	286	10.8	634	14.6
3) Identical end	210	9.4	283	10.7	655	15.1
4) Overlap	10	0.4	20	0.7	201	4.6
5) No overlap	4	0.2	26	1.0	207	4.8

For the *Y. pestis* CO92 genome, >60% of the genes were identical across the two annotations and another ∼30% had identical start or end positions. In total, we found that >95% of the genes as well as the CDSs overlapped across the two prediction methods. Whereas 4.8% of the genes predicted by AGeS were unique, a total of 154 genes (3.7%) predicted by the Sanger Institute were missing in the AGeS annotation. All 68 tRNA genes predicted by AGeS were identical to those predicted by the Sanger Institute, and all 19 rRNA gene predictions overlapped (>96% length overlap), although only 6 rRNA gene predictions were identical in terms of the start and end locations. Annotation comparisons indicated larger differences for the *Y. pestis* CO92 completed genome than for the two draft genomes. These differences could be attributed to the more extensive studies performed in this genome and the frequent annotation updates since it was first sequenced in 2001 [25].

We also compared the annotations at the protein level by contrasting the enzyme functions predicted by the CatFam enzyme profile database with those provided by the other three prediction methods using Enzyme Commission (EC) numbers [37] as the metric for these comparisons. Table 4 shows that, for the *S. hominis* SK119 draft genome, CatFam assigned EC numbers for 515 genes (or 24% of the annotated CDSs), whereas JCVI assigned EC numbers to 565 genes (or 26%). Of these enzymes, 413 overlapped, of which 379 (92%) had identical EC number annotations. It should be noted that for enzymes that had multiple EC number predictions, we considered an identical match when any of the predicted EC numbers matched between the two annotations. We found similar results for the other two genomes, where >81% of the enzymes overlapped and >90% of those had identical EC numbers (Table 4).

**Table 4:** Comparison of enzyme protein function (EC number) predictions between AGeS and other annotation methods for the three analyzed genomes.

	*S. hominis* SK119	*S. aureus* subsp. *aureus* TCH60	*Y. pestis* CO92
No. of enzymes	515 (AGeS) and 565 (JCVI)	562 (AGeS) and 583 (BCM)	833 (AGeS) and 836 (Sanger)
No. of overlapping enzymes	413	459	671
No. of enzymes with multiple EC numbers	36 (AGeS) and 18 (JCVI)	43 (AGeS) and 0 (BCM)	64 (AGeS) and 22 (Sanger)
No. of overlapping enzymes with identical EC numbers	379	437	606

AGeS, Annotation of microbial Genome Sequences; JCVI, J. Craig Venter Institute; BCM, Baylor College of Medicine; EC, Enzyme Commission.

### Visualization

As discussed earlier, to support the visualization of the annotated genomes, we incorporated GBrowse [4], an open source genome browser, into the AGeS system. An example of such visualization is provided in Document S2.

## Discussion

The accuracy of the annotations reported by the AGeS system depends on the quality of the sequenced reads and assembled contigs, as well as on the accuracy of the predictions of its individual bioinformatics tools. The presented comparisons of AGeS annotations against three annotations systems (JCVI, BCM, and Sanger Institute) indicated differences, which primarily arose from the different annotation tools used in the different systems. For example, the annotation of one of the two draft genomes performed at BCM used resources available from the Enteropathogen Resource Integration Center [38]. The other draft genome was annotated at JCVI using their annotation engine [8], which involves many tools, such as BLAST-Extend-Repraze [39], HMMER [40], RFAM [41], and InterPro [42]. In the original annotation of *Y. pestis* CO92 [25], the Sanger Institute used ORPHEUS [43], WUBLAST [44], and FASTA [45] for predicting protein-coding regions and some InterPro databases for function annotation. In addition, *Y. pestis* CO92 is a widely studied and extensively curated genome, where automated annotation tools served only as a first step. Despite these methodological variations, our annotations are in general agreement with the other annotations, as demonstrated by a >94% overlap in the number of identified genes and a significant number of identical features, such as the number of rRNA and tRNA genes, for both completed and draft genome sequences. Although, in general, the assessment of automated function prediction tools is complicated by the different ontologies used in the different classification systems and the lack of “gold standards” [46], comparisons based on EC numbers showed a very good agreement in the pairwise assessment of enzyme predictions between AGeS and the other annotation systems, with each assessment indicating a >90% agreement in the predicted EC numbers.

The current implementation of AGeS for microbial genome annotation has some limitations that shall be addressed in future releases. First, its scope is limited to bacterial genomes. Viral genome annotation requires specialized tools, such as GATU [47], and we are working on their integration into AGeS. Moreover, AGeS input is limited to sequences that are generated from a single genome and thus cannot be used for clinical and metagenomic samples. Third, features are annotated using independent tools and are reported without any filtering, which may lead to unrealistic feature overlap. Post-processing, which takes into account prediction reliability and prior information, will be enhanced to resolve ambiguities, such as those arising in the case of RNA and CDS overlap. Finally, the computational performance of the overall annotation pipeline can be improved by further optimization of the parallel implementations of the individual component tools.

### Conclusions

We have developed a fully integrated, high-performance software system, AGeS, which annotates genomic sequences and assigns function(s) to the predicted protein-coding regions for completed and draft bacterial genomes. Unlike Web servers with similar functionality, AGeS is a standalone system and users can employ their own resources and high-performance computing assets to process, store, and analyze data locally. Although, to date, the focus has been limited to sequence annotation and restricted to bacterial genomes, AGeS has been designed for easy extensibility and future incorporation of different genome annotation and analysis methods whenever they become mature and available. We are currently developing specialized tools and databases for expanding AGeS to the annotation of viral genomes. We also plan on expanding AGeS to include the capability to identify and characterize bacterial and viral pathogens from purified and clinical samples as well as the ability to perform comparative genomic analyses.

AGeS is freely available for download from its home page, http://www.bhsai.org/ages.html, and only requires the availability of Linux operating system. All software tools integrated into AGeS are incorporated during its installation process.

## Supporting Information

Document S1 AGeS User and Installation Manual.
(PDF)

Document S2 AGeS Exemplar Visualization.
(PDF)

GenBank File S1 AGeS annotations for the draft genome of *S. hominis* SK119.
(GBK)

GenBank File S2 AGeS annotations for the draft genome of *S. aureus* subsp. *aureus* TCH60.
(GBK)
