# Supplementary material for: AGeS: A Software System for Microbial Genome Sequence Annotation
Source: PLoS One. 2011 Mar 7;6(3):e17469. doi: 10.1371/journal.pone.0017469 (PMC3049762; doi:10.1371/journal.pone.0017469)
Supplement: Document S1 — AGeS User and Installation Manual. (PDF) [file pone.0017469.s001.pdf]

BHSAI

Biotechnology HPC Software Applications Institute

AGeS

Annotation of microbial Genome Sequences

User and Installation Manual

January 2011

Table of Contents

Introduction 3

Installing AGeS 5

Starting an AGeS Session 12

AGeS GUI Overview 13

- Sequence Management 13
- Annotation Management 15
- Visualization 15

1 Introduction

The cataloguing and analysis of microbial genomes sequenced using next-generation technologies opens new avenues for screening unknown microbes and analyzing their genetic diversity. For such applications, the analysis of sequenced genomes needs to be rapid, high-throughput, fully automated, integrated, and readily accessible to the intended users. To address this need, we have developed the Annotation of microbial Genome Sequences (AGeS) software system, which incorporates publicly available and in-house-developed bioinformatics programs and databases, many of which are parallelized for high-throughput performance.

AGeS performs gene and protein annotation for bacterial genomes using an integrated software pipeline. The input to AGeS is a multi-FASTA file containing contigs generated by high-throughput sequencing. AGeS analyzes these contigs and locates genomic regions that code for proteins, RNA, and other genomic elements by using a set of tools, such as Glimmer [1], RNAmmer [2], and TRF [3], through the Do-It-Yourself Annotation (DIYA) framework [4]. The identified protein coding regions are then annotated using high-throughput protein function annotation methods implemented in the in-house-developed PIPA pipeline [5]. The output of an AGeS run consists of annotated sequences. These annotated sequences are visualized using GBrowse [6], which is fully integrated into the AGeS pipeline. The results can also be downloaded as a GenBank format file for further analysis. Several features make AGeS a useful tool for scientists that need high-throughput annotation of their genomic sequences:

- fully automated annotation of completed and draft bacterial genomes performed by combining the DIYA framework with the PIPA protein function annotation pipeline;
- annotations compliant with the *de facto* standards, i.e., Minimum Information About a Genomic Sequence (MIGS) [7] for genomic sequences, and Gene Ontology [8] for protein function annotations;
- user-friendly visualization based on the familiar open-source genome browser GBrowse; and
- high-throughput annotation accomplished through the efficient use of high-performance computing.

Figure 1-1 shows the system architecture of AGeS. It comprises of a Web application server (AGeS server) that provides an easy-to-use GUI accessible via a Web browser, an embedded relational database management system for storing sequences and other job-related data, and a high-throughput software pipeline for the annotation of input genomes. The AGeS server and annotation pipeline can be installed on either a standalone Linux computer or a Linux cluster using the step-by-step instructions provided in the next chapter “Installing AGeS.” Once the software is installed, multiple users can access the AGeS GUI via the AGeS server using standard Web browsers. The AGeS GUI provides three main functions to the users: (*i*) sequence management for uploading and manipulating genomic sequences; (*ii*) job submission for running the annotation pipeline; and (*iii*) graphical visualization of the annotated sequences with GBrowse. The AGeS server uses a workflow manager module to guide the entire lifecycle of the user’s job from the input sequence upload to the visualization of the annotated sequence.

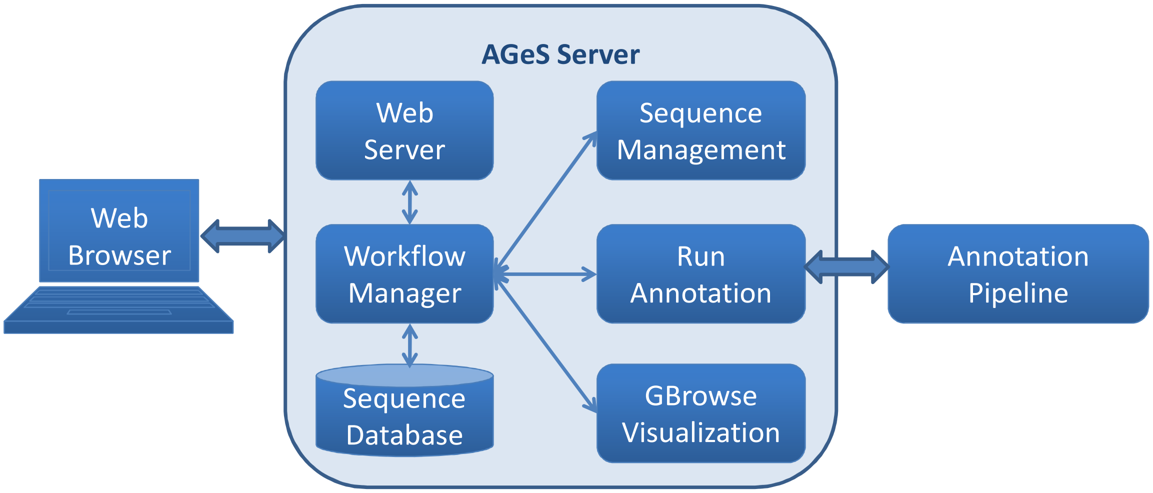

Figure 1-1. AGeS System Architecture

AGeS Requirements: The only requirement for installing AGeS is a Linux operating system. Red Hat Enterprise Linux 5.x or CentOS 5.x distributions of Linux operating system is recommended for installing AGeS. Minimum hardware requirement to run AGeS includes an Intel Pentium 4 2.8 GHz processor, 4 GB RAM, and 150 GB free space on a storage device. A recommended hardware configuration includes an Intel Xeon 2.8 GHz quad-core processor, 8 GB RAM, and 500 GB free storage space. AGeS can be installed in a standalone Linux computer (with single or multiple cores) or a Linux cluster. When run on a multi-core Linux computer or a Linux cluster, AGeS supports OpenMPI for parallel execution and PBS for batch submission. All software tools used by AGeS are installed during the AGeS installation process described in the next chapter “Installing AGeS.”

References:

[1] Delcher AL et al., Bioinformatics. 2007; 23(6):673-9.

[2] Lagesen K et al., Nucleic Acids Res. 2007; 35(9):3100-8.

[3] Benson G, Nucleic Acids Res. 1999; 27(2):573-80.

[4] Stewart AC et al., Bioinformatics. 2009; 25(7):962-3.

[5] Yu C et al., BMC Bioinformatics. 2008; 9:52.

[6] Donlin MJ, Curr Protoc Bioinformatics. 2007; Chapter 9:Unit 9.9.

[7] The minimum information about a genome sequence (MIGS) specification. Nat Biotechnol. 2008; 26(5):541-7.

[8] The Gene Ontology Consortium. Nat Genet. 2000; 25(1):25-9.

2 Installing AGeS

The AGeS source code is available at http://www.bioanalysis.org/downloads/ages.tar.gz. It is freely available under a BSD license. It can be installed using the step-by-step instructions detailed below. These instructions will work unmodified on the Red Hat Enterprise Linux 5.5 or CentOS 5.5 Linux distributions. If you are using another Linux distribution, use these instructions as a guide and adapt to your particular distribution as necessary.

This installation guide assumes that both the GUI and the pipeline will be installed on the same host. If the GUI and the pipeline are installed on different hosts, Steps 1 and 2 are required on the host that is running the GUI, and Steps 1 and 3 are required for the host that is running the pipeline. If the pipeline is running on a cluster, follow Step 4 for configuration of the cluster.

Steps for installing AGeS:

1. Common requirements for AGeS GUI and pipeline

1.1. Download the AGeS tar ball from http://www.bioanalysis.org/downloads/ages.tar.gz. After extracting the tar ball you will see the following directory structure:

```
ages
|-- INSTALL
|-- ages_run
|-- anna_env
|-- bin
|   |-- diya-genbank2gff3.pl
|   |-- jms_local
|   |-- local_book_keeping.sh
|   `-- serialsubmit
|-- conf
|   |-- diya.conf
|   `-- diya_repeat.conf
|-- data
|   |-- UniRef50
|   `-- rfam_dir
|-- diya_env
|-- lib
|   |-- anna.jar
|   `-- org
|       `-- bhsai
|           `-- ages
|               `-- annotation
|                   `-- resources
|                       |-- annotation.properties
|                       |-- diya_default.properties
|                       |-- gbrowse_default.properties
|                       |-- gbrowse_gff.template
|                       |-- pipa_default.properties
|                       |-- post_processing.properties
|                       `-- pre_processing.properties
```

```
|-- mpi_env
|-- pipa_env
|-- rungui.sh
|-- results
|   `-- ages_data
|-- software
|   `-- RepeatFinder
|       |-- RepeatFinder.pl
|       |-- SeqRepeat.pm
|       `-- trf400.linuxAMD64.exe
|-- src
|       |-- data
|       |-- java
|       |-- sql
|       `-- web
`-- web
        |-- data
        |-- lib
        |-- logs
        |-- runserver.sh
        `-- webapps
              |-- ages
              |-- ROOT
              `-- vis
```

1.2. Set the environment variable $AGES_HOME to the ages directory.

1.3. Install Java: JDK 1.6(+) is required to run AGeS GUI. Download and install Java (JDK1.6+) from the Oracle Web page (http://www.oracle.com/technetwork/indexes/downloads/index.html). Setup the $JAVA_HOME environment variable and point it to the JDK installation location.

Example:

1) Install JDK at /home/test/software/jdk1.6.0_22
2) Add the following line to your "~/.bashrc" file

```
export JAVA_HOME=/home/test/software/jdk1.6.0_22
export PATH=$JAVA_HOME/bin:$PATH
```

3) Confirm your Java installation by running "java -version"

1.4. Install Perl and required modules: Perl version 5.10(+) is required for running the AGeS GUI. We recommend manually installing Perl under your home directory in order to avoid conflicts with your system-wide Perl. In addition to Perl, the following Perl modules are required by the AGeS visualization component:

CGI	(2.56 or higher)
GD	(2.07 or higher)

Note that libgd 2.0.28+ is required for the GD module. If your system does not have libgd installed, you can download it at http://www.libgd.org/releases/gd-latest.tar.gz and install it (root access required).

CGI::Session	(4.03 or higher)
DBI	(any version)
DBD:mysql	(any version)

Note that the packages mysql, perl-DBI, perl-DBD-MySQL should be installed in advance. You can use yum to install them (root access required).

Digest::MD5	(any version)
Text::Shellwords	(any version)
Class::Base	(any version)
XML::Parser	(any version)

XML::Writer	(any version)
XML::Twig	(any version)
XML::DOM	(any version)
LWP	(any version)
IO::String	(any version)
MOBY	(any version)
Data::Stag	(any version)

Example:

1) Download Perl from http://www.perl.org/get.html
2) Create the Perl installation directory manually:
```
mkdir -p /home/test/software/perl-5.12.2/bin
```
3) Untar the source package and run the following commands:
```
sh Configure -Dprefix=/home/test/software/perl-5.12.2  (choose default options while prompted)
make
make test
make install
```
4) Add the following lines to your "~/.bashrc" file:
```
export PERL_HOME=/home/test/software/perl-5.12.2
export PATH=$PERL_HOME/bin:$PATH
```
5) Use CPAN to install the following additional modules:
```
perl -MCPAN -e 'shell'
install CGI
install GD
install CGI::Session
install DBI
install DBD::mysql
install Digest::MD5
install Text::Shellwords
install Class::Base
install XML::Parser
install XML::Writer
install XML::Twig
install XML::DOM
install LWP
install IO::String
install MOBY
install Data::Stag
quit
```

1.5. Install BioPerl and Bio::Graphics: BioPerl 1.6.1 is required for running AGeS. Download BioPerl from http://www.bioperl.org and install it. The module Bio::Graphics (1.97+) and Bio::Das (any version) are required.

Example:

1) Download BioPerl:
```
wget http://bioperl.org/DIST/BioPerl-1.6.1.tar.gz
```
2) Untar the package file and run the build script:
```
tar zxvf BioPerl-1.6.1.tar.gz
cd BioPerl-1.6.1
perl Build.PL (choose default options)
./Build install
```
3) Check the BioPerl installation by running the following command:
```
perl -MBio::Root::Version -e 'print $Bio::Root::Version::VERSION,"\n"'
```
4) Install the Bio::Das module:
```
perl -MCPAN -e 'shell'
install Bio::Das
```

quit

5) Install the Bio::Graphics module:

```
perl -MCPAN -e 'shell'
install Bio::Graphics
quit
```

6) Add the following lines to your "~/.bashrc" file:

export PERL5LIB=$PERL_HOME/lib/site_perl/5.12.2
export PERL5LIB=$PERL5LIB:$PERL_HOME/lib/site_perl/5.12.2/Bio ***(important)***

2. Steps for installing the AGeS GUI

2.1. Install GBrowse (1.7): The AGeS GUI makes use of GBrowse for the visualization module. Go to http://sourceforge.net/projects/gmod/files/ and download the Generic-Genome-Browser-1.70.tar.gz under the GBrowse-1.70 section. Untar the package and follow the installation instructions. Since AGeS has an embedded Web server, it does not use Apache. During the installation of GBrowse, you can specify a temporary directory when you are asked for Apache path.

Example:

1) Download GBrowse 1.7:

wget http://sourceforge.net/projects/gmod/files/Generic%20Genome%20Browser/GBrowse-1.70/Generic-Genome-Browser-1.70.tar.gz/download

2) Untar the package:

```
tar zxvf Generic-Genome-Browser-1.70.tar.gz
cd Generic-Genome-Browser-1.70
```

3) Install GBrowse:

perl Makefile.PL

Use the following options if you do not have an Apache installed, otherwise change them based on your system configuration:

```
Apache root directory (enter '' for none)?
Apache conf directory? [/etc/httpd/conf] /tmp
Apache htdocs directory? [/var/www/html] /tmp
Apache cgibin directory? [/var/www/cgi-bin] /tmp
GBrowse root for static files? [gbrowse]
make
make install UNINST=1
```

2.2. Configure Email: AGeS uses “localhost” as the SMTP server to send out notification emails. You can change the SMTP server configuration and the “mail from” address. Change the “jbpm.mail.smtp.host” and “jbpm.mail.from.address” properties in the “$AGES_HOME/web/webapps/ages/WEB-INF/classes/jbpm.cfg.xml” configuration file according to your preferences.

2.3. Run the GUI: Once all the required modules are installed, make sure that the required environment variables are set (PATH, JAVA_HOME, PERL_HOME, and PERL5LIB). Go to the ages root directory and run the script "rungui.sh." It will bring up a Jetty Web server that listens on port 9000. Open a Web browser and enter the URL "http://localhost:9000/ages" to access the Web GUI for AGeS. The Web server can be also accessed from another computer provided that port 9000 is not blocked by the firewall. In this case, use your hostname or IP address to replace the "localhost" in the URL. http://<your_host_name or your host IP address>:9000/ages.

2.4. You can run a demo annotation task though the GUI. The demo uses a pre-loaded GenBank file as the annotation output for the demonstration purpose. In order to run a real annotation, you have to install and configure the annotation pipeline using Step 3.

2.5. Customize Database (Optional): The AGeS distribution package uses Apache Derby as its default database engine. If you intend to deploy the application in a production server with a large user base, we recommend using a standalone database server, such as PostgreSQL. The AGeS package has been tested on PostgreSQL 8.3. Please follow the steps below to configure your system for PostgreSQL:

2.5.1. Create a database called "ages".

2.5.2. Create tables using the SQL script located at src/sql/postgresql/AgesPkg-Creation-postgres.sql.
2.5.3. Change the database configuration file located at web/webapps/ages/WEB-INF/classes/db.properties with the following information:

```
db.type=postgres
db.driver.classname=org.postgresql.Driver
db.username=<the database user name>
db.password=<the password for database user>
db.url=jdbc:postgresql://<your database server host name>:<database server port>/ages
```

2.5.4. Run the GUI as described in Step 2.3 above.

3. Steps for installing AGeS pipeline

The AGeS pipeline was tested using JDK 1.6, Perl 5.10.1 and BioPerl 1.6. If you followed the previous steps you should already have the required software library installed.

3.1. Install and configure DIYA: You can download DIYA from its SourceForge page at http://sourceforge.net/projects/diyg/ and Install it at $AGES_HOME/software/diya. To run DIYA as part of AGeS you need to install the following software in the $AGES_HOME/software directory:

Blast	(ftp://ftp.ncbi.nih.gov/blast/executables/release/)
hmmer	(http://hmmer.janelia.org/)
Glimmer	(http://www.cbcb.umd.edu/software/glimmer/glimmer302.tar.gz)
infernal	(http://infernal.janelia.org/)
MUMmer	(http://mummer.sourceforge.net/)
tRNAscan	(http://lowelab.ucsc.edu/tRNAscan-SE/)
rnammer	(http://www.cbs.dtu.dk/services/RNAmmer/)

After installation of individual DIYA software components, create the following symbolic links in the $AGES_HOME/software directory to the appropriate directories:

```
blast-->blast-2.2.19
hmmer-->hmmer-2.3.2
glimmer-->Glimmer3.02
infernal-->infernal-1.0.2
MUMmer-->MUMmer3.20
tRNAscan-SE-->tRNAscan-SE-1.23
rnammer-->rnammer-1.2
diya-->diya-1.0
```

As part of DIYA, download UniRef50 database from the EBI Web site ftp://ftp.ebi.ac.uk/pub/databases/uniprot/uniref/uniref50/uniref50.fasta.gz and install it in $AGES_HOME/data/UniRef50/ using the following command:

```
ages/software/blast-2.2.19/bin/formatdb -i uniref50.fasta -p T -o T -t uniref50 -n uniref50
```

You can parallelize DIYA using mpiBLAST. Please refer to Step 4.1 for installing and setting up mpiBLAST for DIYA. If using mpiBLAST, make appropriate changes in the data formatting procedure.

Configuration of the DIYA components can be controlled by the file $AGES_HOME/conf/diya_repeat.conf. To run the correct version of these components, change the paths appropriately in the configuration file $AGES_HOME/conf/diya_repeat.conf. If using mpiBLAST, make appropriate changes in the DIYA configuration file.

DIYA also uses the property file diya_default.properties located in the $AGES_HOME/lib directory to specify the location of temporary files. These properties are overridden by the properties in the annotation.properties file.

3.2. Install and configure PIPA: Download PIPA from http://www.bhsai.org/downloads/PIPA.tar.gz and install it in the $AGES_HOME/software/PIPA directory. Make sure you set the following environment variables:

```
export PIPA_HOME=AGES_HOME/software/PIPA
export PIPA_WRAP=AGES_HOME/software/PIPA/src/Profann/dsrc/pipa_submission_wrapper.pl
export MERGE_GBK_GFF=AGES_HOME/software/PIPA/src/Profann/misc/mergePipaGff2Genbank.pl
export MPI_PIPA=AGES_HOME/software/PIPA/src/Profann/App/MPIProfannBatch.pl
```

export IPRSCAN_HOME=$PIPA_HOME/software/iprscan

PIPA also uses the property file pipa_default.properties located in the $AGES_HOME/lib directory to specify the location of temporary files. These properties are overridden by the properties in the annotation.properties file. You can also override the tools to run within PIPA, the default parameters of these tools and whether to run PIPA in MPI or SERIAL. To run PIPA in MPI mode you will also have to make sure that mpirun and jobmanager parameters are set in the $PIPA_HOME/config/pipa_user.config configuration file.

3.3. Set environment variables: Set the environment variable $AGES_HOME to the ages directory. Make sure you have the permission to execute $AGES_HOME/software/RepeatFinder/RepeatFinder.pl, $AGES_HOME/bin/diya-genbank2gff3.pl, $AGES_HOME/bin/jms_local, $AGES_HOME/bin/local_book_keeping.sh, and $AGES_HOME/bin/serialsubmit. Add $AGES_HOME/lib, and jar files anna.jar, biojava.jar, activemq-all-5.2.0.jar, bytecode.jar and commons-cli-1.2.jar in $AGES_HOME/lib to CLASSPATH. Also set the environment variables for all the dependencies (e.g., PIPA, DIYA, Pipeman, OpenMPI, etc.) as required.

3.4. Running Annotation pipeline through GUI: The AGeS GUI interfaces with the pipeline through the *_env files. Make sure your default shell is bash. Make appropriate changes in the files anna_env, mpi_env, pipa_env, and diya_env.

4. Steps to Run AGeS on a Cluster (Optional)

The AGeS pipeline can be configured to run on clusters for high-throughput annotation. Parallelization can be achieved through message passing interface (mpiBLAST for DIYA and MPI for PIPA). The pipeline can also be invoked through a PBS batch submission system. Therefore, there are three operation modes for AGeS: serial, MPI, and PBS.

4.1. Set up mpiBLAST for DIYA: mpiBLAST is required to run DIYA in parallel mode. The following steps explain how to set up mpiBLAST for DIYA:

- 4.1.1. Download and install mpiBLAST from http://www.mpiblast.org/
- 4.1.2. Get uniref50 database from ftp://ftp.ebi.ac.uk/pub/databases/uniprot/uniref/uniref50/uniref50.fasta.gz
- 4.1.3. Format uniref50 database using mpiformatdb script, e.g., /mpiblast-1.5.0-pio/bin/mpiformatdb -N 32 -i uniref50.fasta -p T -o T -t uniref50 -n uniref50
- 4.1.4. Provide path to mpiBLAST in $AGES_HOME/bin/batchblast.
- 4.1.5. Uncomment batchblast <parser> for blastp::CDS in diya.conf and diya_repeat.conf and comment blastall <parser>.
- 4.1.6. Change the properties file located at <root>/src/java/org/bhsai/ages/annotation/resources/diya_default.properties:
  mode=MPI *(if not using batch submission system)*

4.2. Set up MPI for PIPA

- 4.2.1. PIPA can run in parallel mode using MPI. Consult with your cluster administrator and make sure OpenMPI or MPICH is installed on the cluster. Please refer to the installation documentation in the PIPA package regarding how to compile and execute PIPA using MPI.
- 4.2.2. Change the properties file located at <root>/src/java/org/bhsai/ages/annotation/resources/pipa_default.properties:
  mode=MPI *(if not using a batch submission system)*

4.3. Change the configurations to support PBS (if using PBS batch submission system)

AGeS supports the PBS batch submission system. Two configuration files are required to be changed in order to submit an AGeS job:

- 4.3.1. In the $AGES_HOME/src/java/org/bhsai/ages/annotation/resources/diya_default.properties file:
  mode=PBS
  Make changes to “pbs.*” properties as needed.
- 4.3.2. In the $AGES_HOME/src/java/org/bhsai/ages/annotation/resources/pipa_default.properties file:
  mode=PBS
  Make changes to “pbs.*” properties as needed.

4.4. Set up a Java message service tunneling between the computation nodes and the Web server

- 4.4.1. If running the AGeS pipeline in a PBS batch submission system, it is necessary to run the GUI Web server on the same cluster in an interactive session.
- 4.4.2. On the cluster, set an environment variable $WORKDIR to a path accessible from all nodes of cluster.

4.4.3. Create a password-free public/private key pair and store the private key in the $AGES_HOME/conf directory. Add the public key into the authorized_keys file. For more information about public/private key authentication, refer to:

http://hkn.eecs.berkeley.edu/~dhsu/ssh_public_key_howto.html

4.4.4. Change the property file located at $AGES_HOME/src/java/org/bhsai/ages/annotation/resources/annotation.properties:

jms_client=env'AGES_HOME'/bin/jms

3 Starting an AGeS Session

The screenshot in Figure 3-1 shows the entry page of the AGeS system. The user starts AGeS by clicking on the **Enter** button. Unique session IDs identify different users. If you have visited the site before, your session ID will be automatically appended to the URL and your previous data will be automatically loaded.

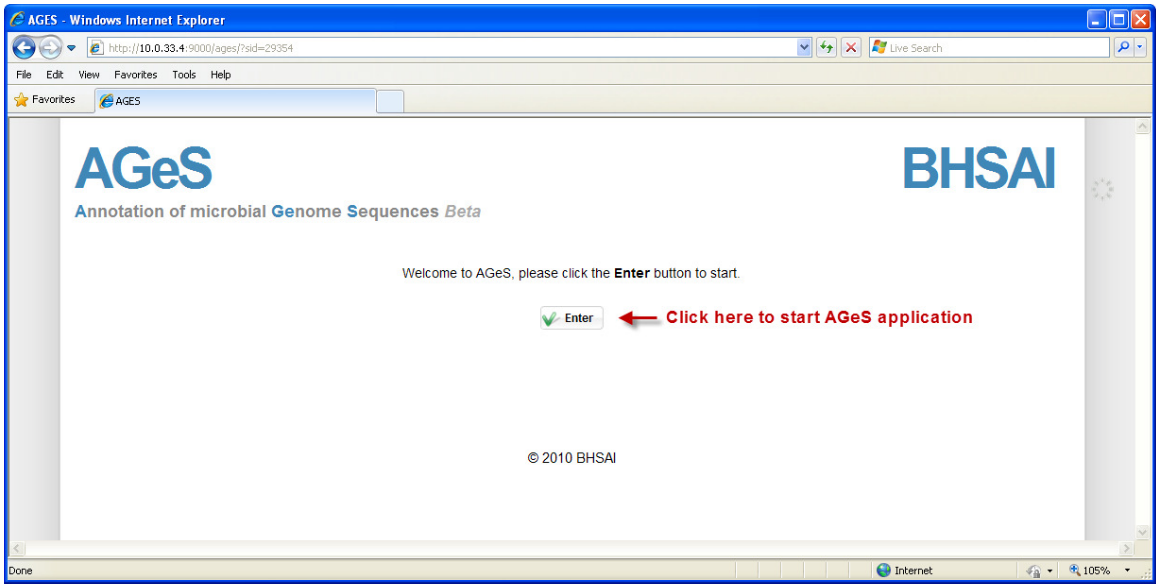

Figure 3-1. Starting an AGeS session

Once the AGeS session is completed, the user can click the **Exit** button on the upper-right side of the page (Figure 3-2) to navigate back to the AGeS entry page.

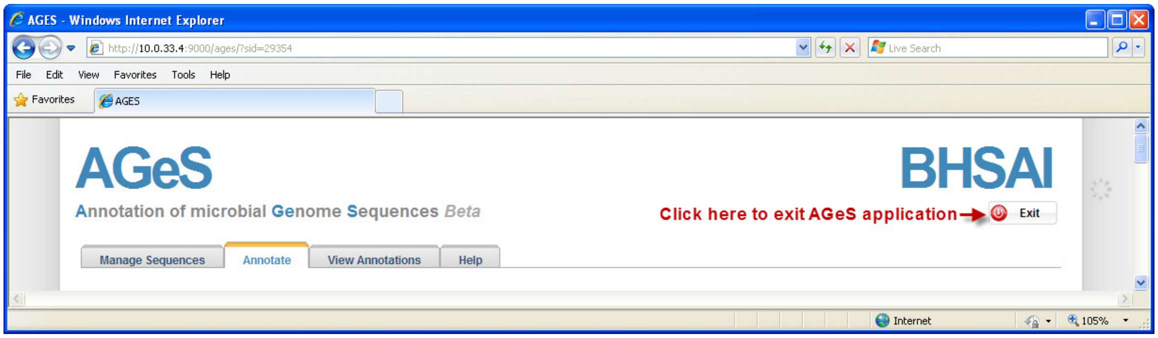

Figure 3-2. Exiting from an AGeS session

4 AGeS GUI Overview

Three main components of the AGeS system are accessed through the three tabs on the AGeS main page: **Manage Sequences**, **Annotate**, and **View Annotations**, as presented in Figure 4-1. The user can upload and store the sequences (contigs in multi-FASTA format) through the **Manage Sequence** tab. The next step is to annotate these contigs, i.e., to find genes, the locations of these genes in the contigs, the proteins encoded by the genes, and the functions of these proteins. These tasks are performed under the **Annotate** tab. Finally, the user can view the annotations using the **View Annotations** tab.

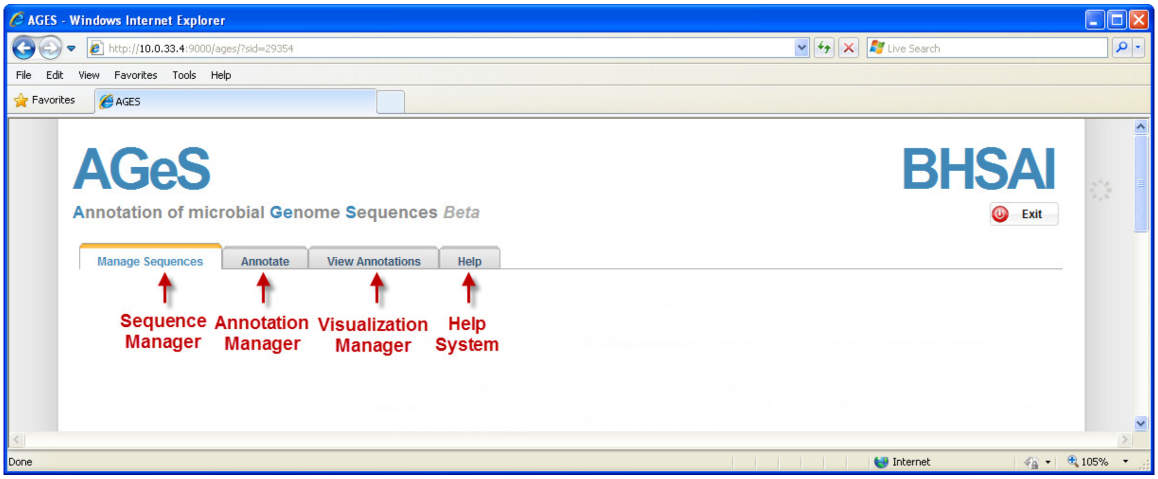

Figure 4-1. Main options in AGeS

Sequence Management

There are two ways to load your sequence into AGeS: copy the contigs (in FASTA format) and paste them in the text box, or upload a FASTA file. The screenshot in Figure 4-2 shows how to copy/paste a sequence segment, and Figure 4-3 shows how to upload a sequence in FASTA format.

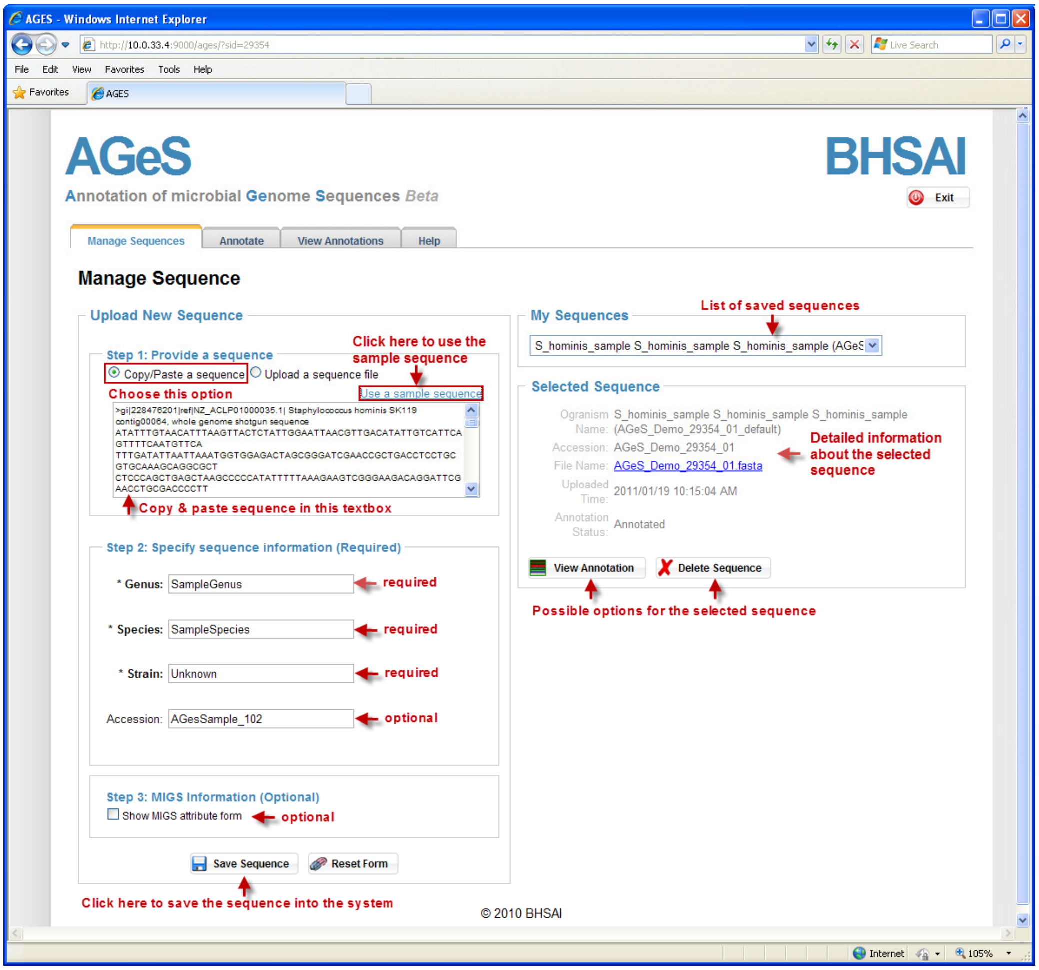

Figure 4-2. Copying/Pasting a sequence in AGeS

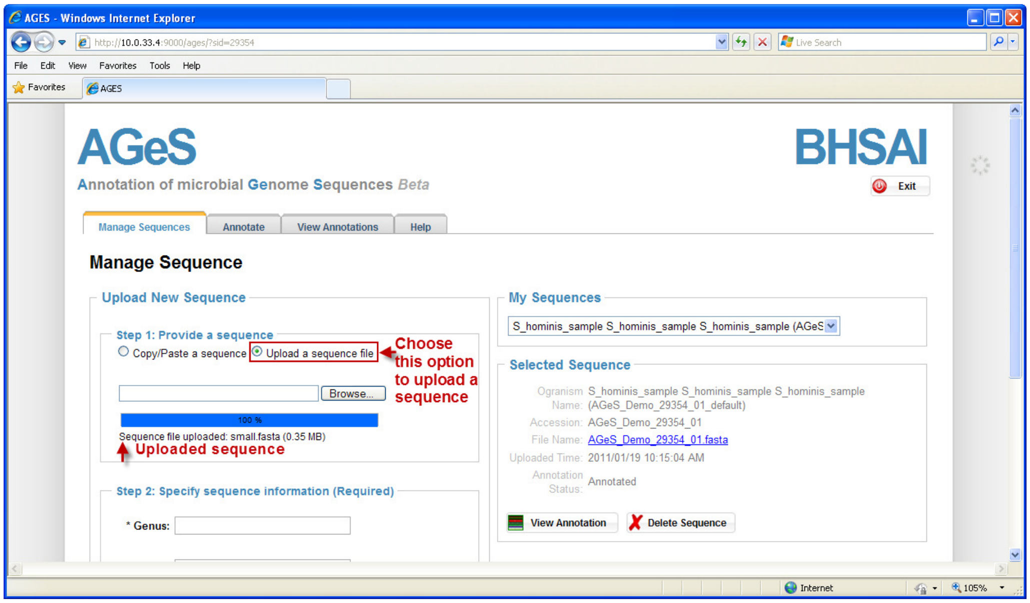

Figure 4-3. Uploading a FASTA-formatted file

Annotation Management

Figure 4-4 shows the **Annotate** tab. The box on the right shows the sequences whose annotations are pending and/or those that have already been annotated, and on the left side are sequences yet to be annotated. The user should select one of these sequences and hit the **Annotate** button.

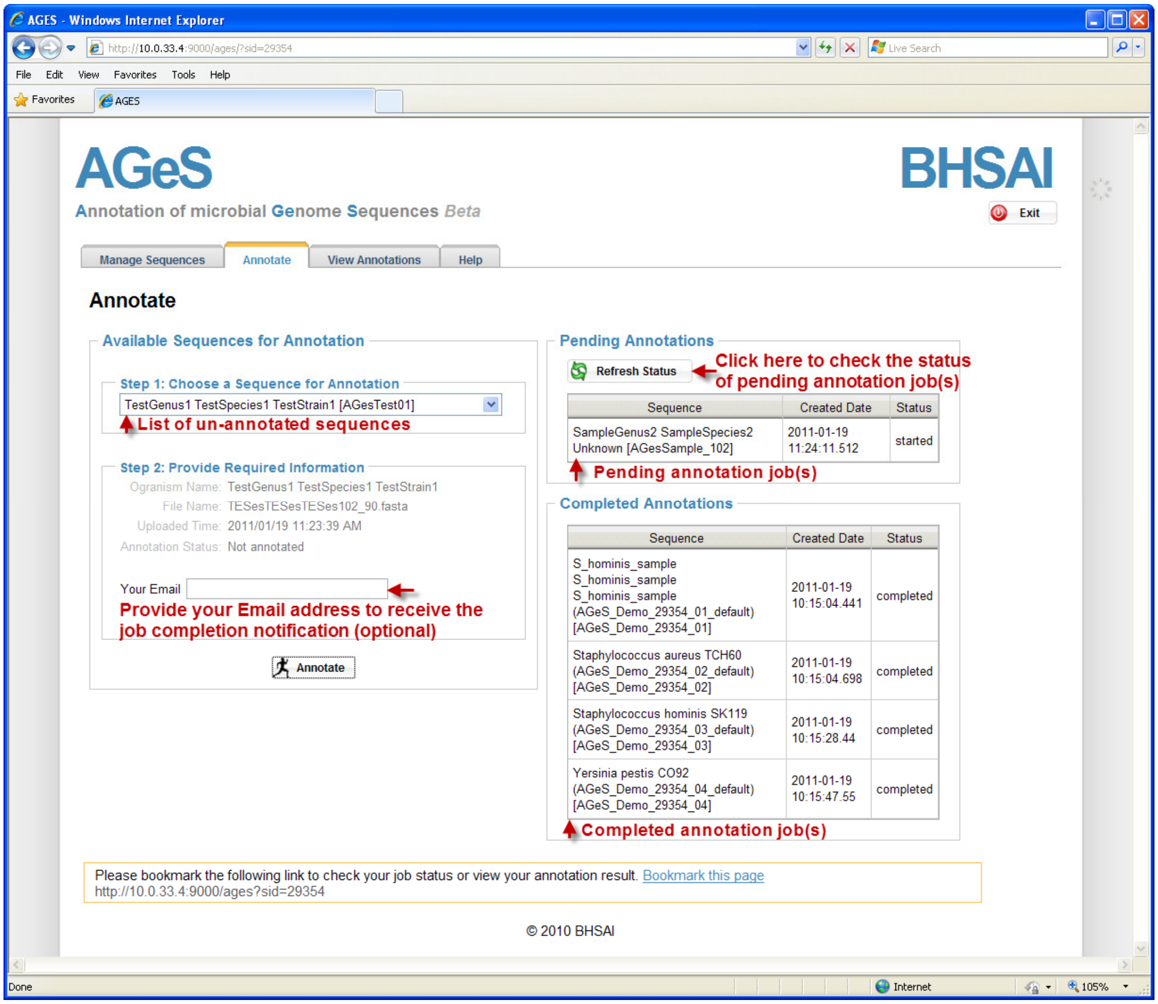

Figure 4-4. Starting annotation

Visualization

The user can visualize the annotation by clicking on the **View Annotations** tab.

The example in Figure 4-5 shows the annotation of three contigs from the *Staphylococcus hominis* SK119 genome spanning a region of 86.5 kbp. The customized view shows the location of contigs, contig boundaries, genes, coding sequences (CDSs), rRNAs, tRNAs, and tandem repeats. The inset shows the zoomed-in view of a 2.2-kbp region with the arrow pointing toward the CDS feature AGES379_20670.t01.

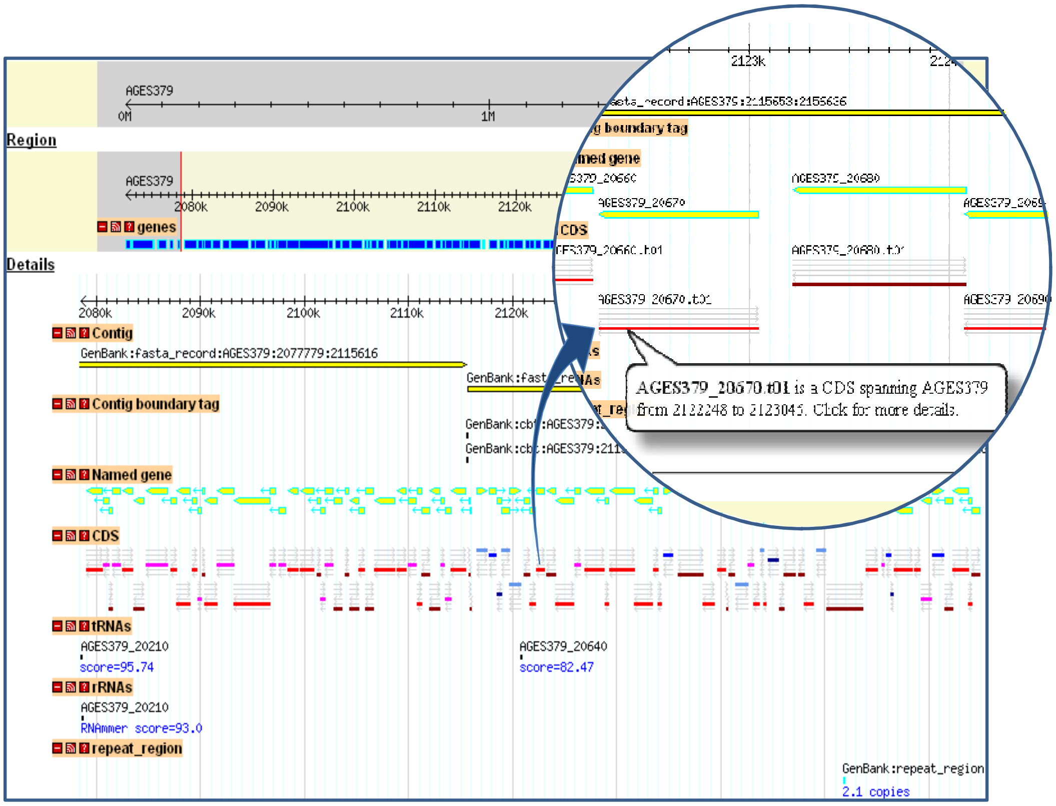

Figure 4-5. Visualization of the annotation results with GBrowse

For questions or problems related to AGeS please contact: ages@bioanalysis.org.
