# Supplementary material for: AGeS: A Software System for Microbial Genome Sequence Annotation
Source: PLoS One. 2011 Mar 7;6(3):e17469. doi: 10.1371/journal.pone.0017469 (PMC3049762; doi:10.1371/journal.pone.0017469)
Supplement: Document S2 — AGeS Exemplar Visualization. (PDF) [file pone.0017469.s002.pdf]

AGeS Exemplar Visualization

Figure 1 shows the GBrowse visualization of an annotated genome, with each of the annotation features shown on a separate track under "Details." The target of this annotation was an 86.5-kbp nucleotide sequence containing three contigs from the *S. hominis* SK119 strain. A large number of genes and CDS features are shown in addition to two tRNAs, one rRNA, and one tandem repeat. These annotations can be downloaded to the user's computer by clicking the "Download GenBank" link on the "View Annotations" page (not shown in the figure) or browsed further using GBrowse. Users can navigate the target genome in GBrowse by scrolling up and down the genome and zooming in and out to change the resolution. For example, the *inset* in Figure 1 shows the zoomed-in view of a 2.2-kbp region. Users can also hide/display specific tracks and customize the appearance of the graphical view according to their needs.

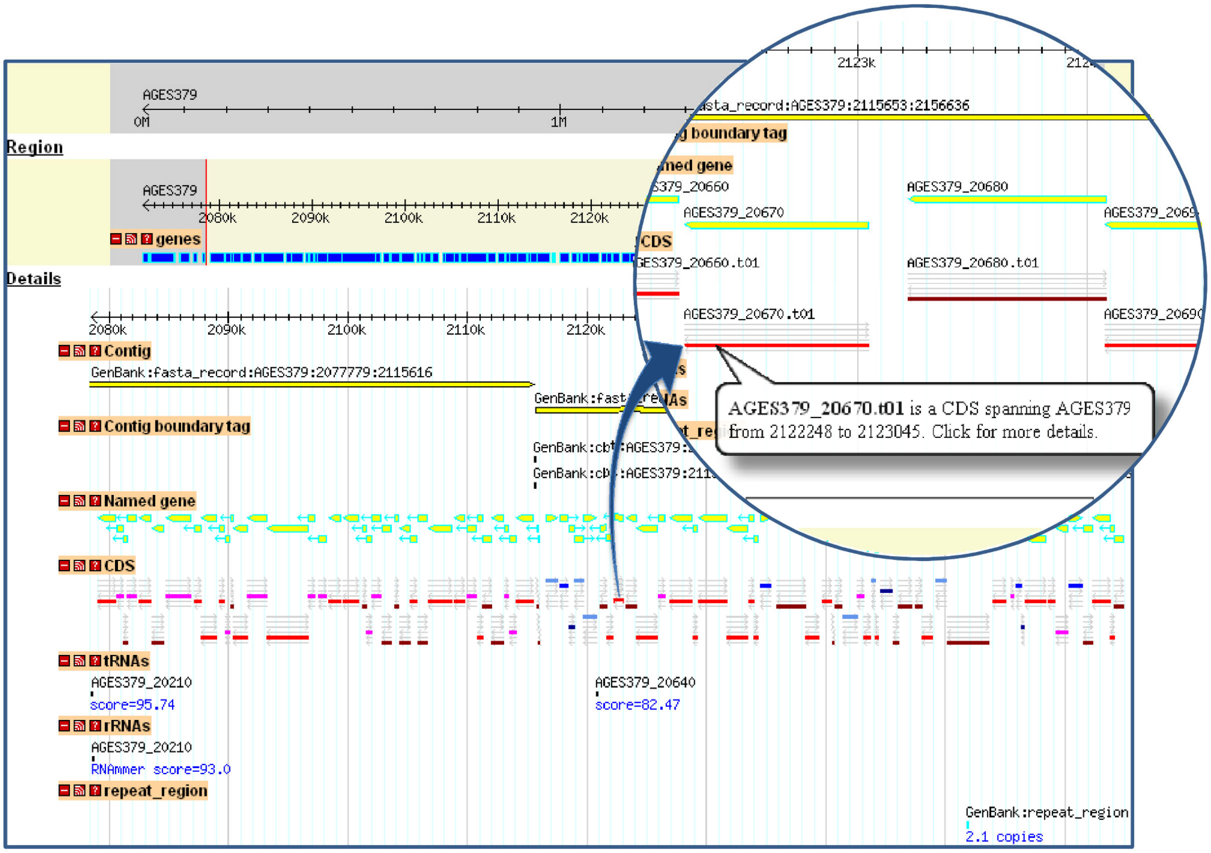

Figure 1. Visualization of the annotation results with GBrowse. This example shows the annotation of three contigs from the *Staphylococcus hominis* SK119 genome spanning a region of 86.5 kbp. The customized view shows the location of contigs, contig boundaries, genes, coding sequences (CDSs), rRNAs, tRNAs, and tandem repeats. The *inset* shows the zoomed-in view of a 2.2-kbp region with the arrow pointing toward the CDS feature AGES379_20670.t01, for which detailed functional annotation is shown in Figure 2.

By clicking on each feature of the graphical display, the user can obtain additional detailed information about the feature. For example, Figure 2 shows the protein function annotation details for the CDS feature AGES379_20670.t01, as highlighted in the *inset* in Figure 1. Function annotation is reported for EC numbers and two GO categories, biological process (P) and molecular function (F). The EC number (5.1.1.3) and the annotation "Glutamate racemase" reported under "product" coincided with those provided by JCVI.

**Name:**	**AGES379_20670.t01**
**Type:**	processed_transcript
**Description:**	score=442
**Source:**	GenBank
**Position:**	AGES379:2122248..2123045 (- strand)
**Length:**	798
**Parent:**	AGES379_20670.t01
**eC_number:**	5.1.1.3
**function:**	GO:0008881 glutamate racemase activity[F]<br>GO:0009252 peptidoglycan biosynthetic process[P]<br>Cytoplasmic
**locus_tag:**	AGES379_20670
**note:**	score=442
**product:**	Glutamate racemase n=28 Tax=Bacillales RepID=MURI_STAS1
**score:**	442
**Parts:**	**Type:** CDS

Figure 2. Protein function annotation. Function annotation for the predicted protein coding region AGES379_20670.t01 is reported with Enzyme Commission (EC) numbers and Gene Ontology (GO) categories [biological process (P), cellular component (C), and molecular function (F)] at various levels of specificity. In this example, the cellular component was not assigned. The most specific annotation reported by the J. Craig Venter Institute for this protein was "Glutamate racemase," which coincided with the "product" annotation reported by AGeS. The corresponding EC number (5.1.1.3) was also the same for both annotations.
